# Synergistic effects of polymyxin and vancomycin combinations on carbapenem- and polymyxin-resistant *Klebsiella pneumoniae* and their molecular characteristics

**DOI:** 10.1128/spectrum.01199-23

**Published:** 2023-10-31

**Authors:** Ozioma Forstinus Nwabor, Arnon Chukamnerd, Pawarisa Terbtothakun, Lois Chinwe Nwabor, Komwit Surachat, Sittiruk Roytrakul, Supayang Piyawan Voravuthikunchai, Sarunyou Chusri

**Affiliations:** 1 Division of Infectious Diseases, Department of Internal Medicine, Faculty of Medicine, Prince of Songkla University, Hat Yai, Songkhla, Thailand; 2 Division of Biological Science, Faculty of Science and Natural Product Research Center of Excellence, Prince of Songkla University, Hat Yai, Songkhla, Thailand; 3 Department of Biomedical Sciences and Biomedical Engineering, Faculty of Medicine, Prince of Songkla University, Hat Yai, Songkhla, Thailand; 4 Faculty of Medicine, Translational Medicine Research Center, Prince of Songkla University, Hat Yai, Songkhla, Thailand; 5 Functional Proteomics Technology Laboratory, National Center for Genetic Engineering and Biotechnology, National Science and Technology Development Agency, Pathumthani, Thailand; 6 Faculty of Science, Center of Antimicrobial Biomaterial Innovation-Southeast Asia and Natural Product Research Center of Excellence, Prince of Songkla University, Hat Yai, Songkhla, Thailand; University of Saskatchewan, Saskatoon, Saskatchewan, Canada

**Keywords:** polymyxins, vancomycin, combination therapy, population analysis profiling, genomic profiling, protein profiling

## Abstract

The emergence and spread of polymyxin resistance, especially among *Klebsiella pneumoniae* isolates, threaten the effective management of infections. This study profiled for polymyxin resistance mechanisms and investigated the activity of polymyxins plus vancomycin against carbapenem- and polymyxin-resistant *K. pneumoniae*. The entire genome sequences of seven isolates were profiled for resistance and virulence determinants. The effects of combination therapy were evaluated using the checkerboard technique, time-kill assay, and population profile analysis. Protein profiles of the isolates treated with monotherapy were compared to that of combination therapy. The whole-genome sequencing data revealed that the isolates harbored β-lactams, carbapenems, aminoglycoside, fluoroquinolones, macrolides, and tetracycline resistance genes, with several virulence-associated genes. The plasmids including the *bla*_OXA-232_-bearing ColKP3 plasmid were also identified in our isolates. Profiling for polymyxin resistance mechanism revealed a missense mutation in the *crrB* gene that resulted in a Q180L variant that conferred a deleterious effect on protein function. The combination assay indicated fractional inhibitory concentration index ranging from 0.31 to 1.13, whereas the time-kill assay demonstrated synergistic log reduction in colony-forming units per milliliter. Furthermore, population analysis profiling using dual antibiotics indicated enhancement in bacterial log reduction at lower antibiotics concentrations, compared to higher concentrations of single polymyxins. For protein profiling, 796 proteins were identified, and 56 and 94 of them were increased and decreased in the combined drug treatment groups, respectively, while other differentially produced proteins were detected in all treatment groups, except for the control group. The results demonstrated that the vancomycin combination might benefit the antimicrobial activities of polymyxins.

**IMPORTANCE:**

This study provides insights into the mechanisms of polymyxin resistance in *K. pneumoniae* clinical isolates and demonstrates potential strategies of polymyxin and vancomycin combinations for combating this resistance. We also identified possible mechanisms that might be associated with the treatment of these combinations against carbapenem- and polymyxin-resistant *K. pneumoniae* clinical isolates. The findings have significant implications for the development of alternative therapies and the effective management of infections caused by these pathogens.

## INTRODUCTION

Global health care is challenged by the increasing emergence and spread of infections caused by antibiotics and multidrug-resistant (MDR) bacteria. In Thailand, medical practitioners are without strict restrictions on antibiotic choice and at liberty to administer antibiotics deemed effective against MDR pathogens. Thus, permutations of antibiotic combinations as last-resort options are on the increase, and several *in vitro* and *in vivo* studies, as well as clinical trials, have demonstrated that antibiotic combination therapies ensure positive outcomes compared to monotherapies.

Although once discontinued for reported cases of toxicity, polymyxins, a group of polypeptide antibacterial compounds, are currently choice options for the management of infections caused by MDR Gram-negative bacteria, especially carbapenem-resistant pathogens (1 – 3). Among the polymyxin class, polymyxins B and E (colistin) are clinically relevant and share structural similarities, except for an amino acid in the L-Dab peptide ring (4). Both also share a similar antimicrobial spectrum, with activity against most Gram-negative bacteria. Nephrotoxicity and neurotoxicity are common concerns associated with polymyxin B and colistin. However, studies have reported lower rates of nephrotoxicity with polymyxin B compared to colistin (5, 6).

The recent emergence of polymyxin resistance among Gram-negative pathogens (7, 8) constitutes a public health emergency and is proposed to mark an end to the antibiotic era (9). Colistin resistance in Gram-negative bacteria is mostly due to acquired mutations in the two-component systems including PhoPQ, CrrAB, and PmrAB (10 – 12). In addition, the emergence of the *mcr*-colistin resistance gene mobilized by transmissible plasmids (13 – 16) promotes the spread and prevalence of polymyxin resistance. The use of multiple antimicrobial agents in combination therapies has been adopted as an interim viable solution to the management of MDR infections. Studies have demonstrated that polymyxins in combination with other antibiotic classes act in synergy against MDR pathogens (7, 17, 18). Various studies have investigated the use of antimicrobial agents ineffective against Gram negative as adjunctive agents to enhance the inactivation of MDR Gram negatives or as resistance modifying agents to attenuate bacterial mechanisms of resistance.

Vancomycin, a bactericidal glycopeptide antibiotic indicated for Gram-positive bacteria, inhibits cell wall biosynthesis by preventing the incorporation of N-acetylmuramic acid- and N-acetylglucosamine-peptide subunits into the peptidoglycan matrix. In addition, vancomycin damages protoplasts by affecting the cytoplasmic membrane through the inhibition of RNA synthesis (19). Although vancomycin is not indicated for the treatment of Gram-negative bacteria, drug repurposing is proposed as a fast and inexpensive strategy for the management of MDR infections. Studies have evinced the *in vitro* synergistic effects of vancomycin as adjunctive therapy in combination with polymyxins for the management of MDR isolates of *Acinetobacter baumannii* and other clinically relevant bacterial strains (20 – 22). We then hypothesized that the combination of vancomycin and polymyxin (B and E) might be used to combat carbapenem- and polymyxin-resistant *K. pneumoniae* clinical isolates. Thus, this study aims to investigate the antibacterial effects of vancomycin plus polymyxin combinations on these pathogens. Resistant mechanisms and possible mechanisms that occurred from these combinations were also studied by whole-genome sequencing and protein profiling, respectively.

## RESULTS

### Antibacterial activity of carbapenems, polymyxin, and vancomycin

Minimum inhibitory concentrations (MICs) of vancomycin after 2-h resazurin exposure ranged from 512 to 1024 µg/mL. With prolonged incubation of the cells, color changes were subsequently observed after 24 h in higher antibiotics concentrations of 2,048 and 4,096 µg/mL, suggesting bacteria re-growth. The MICs of imipenem and meropenem on the isolates were >64 and >128 µg/mL, respectively. The antibacterial effects of antibiotics on the isolates are presented in Table 1. According to Clinical and Laboratory Standards Institute (CLSI), the antimicrobial breakpoint average of *Enterobacterales* describes resistance to imipenem, meropenem, polymyxin B, and colistin as MIC values ≥4 µg/mL (23).

**TABLE 1:** Minimum inhibitory concentrations and combination effects of carbapenem, polymyxins, and vancomycin^*a*^

Isolate code	MIC antibiotics alone (µg/mL)					FICI range			
	IPM	MEM	PMB	CST	VAN	PMB + VAN	Interpretation	CST + VAN	Interpretation
NT03	>64	>128	512	1,024	512	0.50–0.63	SYN/ADD	0.5–0.63	SYN/ADD
NT05	>64	>128	256	1,024	512	0.50–0.64	SYN/ADD	0.38–0.56	SYN/ADD
NT07	>64	>128	512	1,024	512	0.50–1.13	SYN/ADD/IND	0.56–1.13	SYN/ADD/IND
NT08	>64	>128	512	1,024	1,024	0.38–0.56	SYN/ADD	0.31–1.13	SYN/ADD/IND
NT09	>64	>128	512	512	1,024	0.38–0.56	SYN/ADD	0.50–1.13	SYN/ADD/IND
NT10	>64	>128	1024	1,024	512	0.56–0.75	ADD	0.38–1.13	SYN/ADD/IND
NT23	>64	>128	1024	512	512	0.75–1.13	ADD/IND	0.50–0.63	SYN/ADD
ATCC 19606	<128	<128	1	2	>256	ND	ND	ND	ND

^*a*^ NT, Naradhiwas Rajanagarindra Hospital; IPM, imipenem; MEM, meropenem; VAN, vancomycin; CST, colistin; PMB, polymyxin B; SYN, synergy; ADD, additive; IND, indifferent; FICI, fractional inhibitory concentration index; ND, not detected.

### Detection of antimicrobial resistance genes, virulence genes, and plasmid types

**Fig 1:**
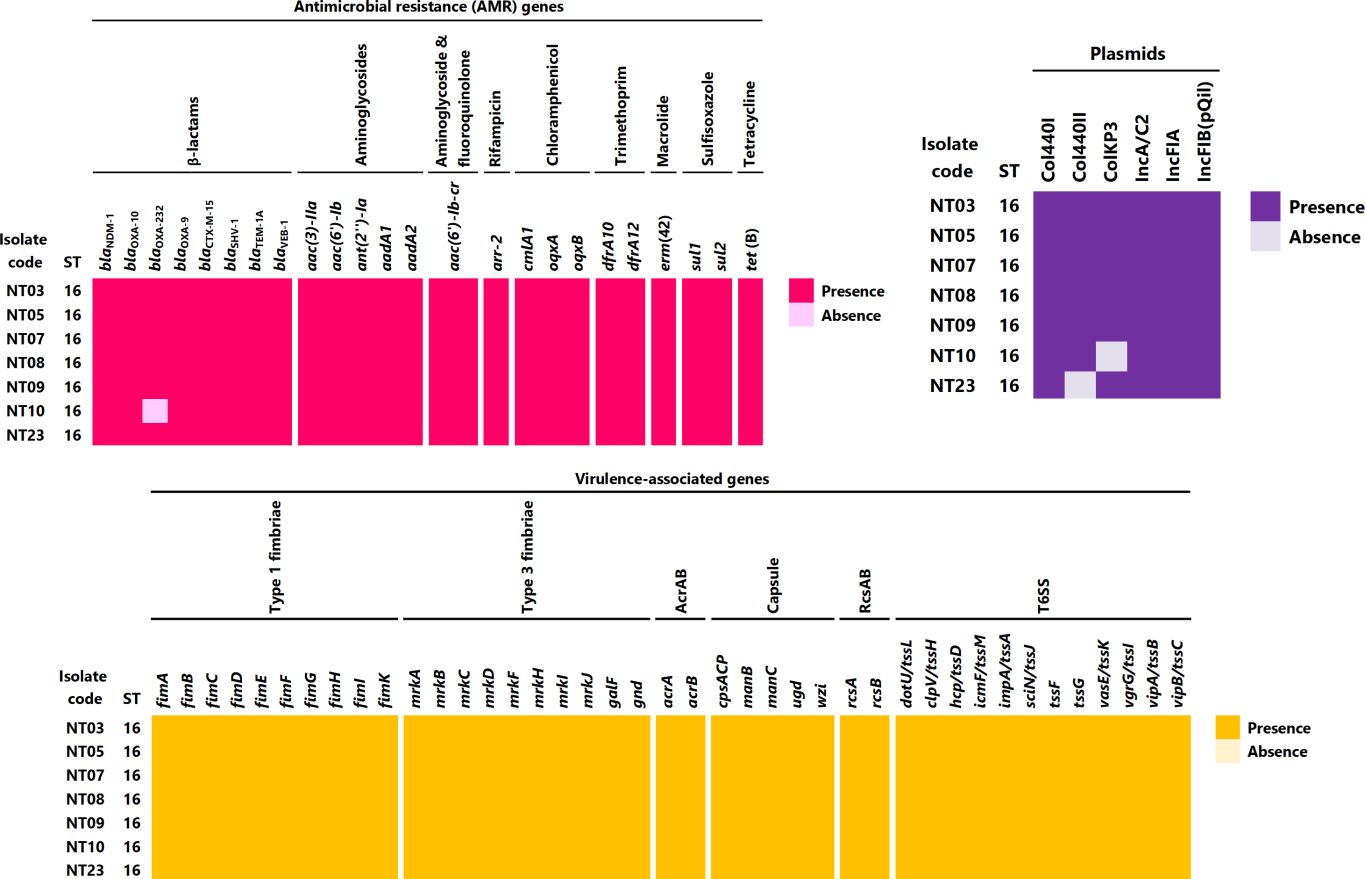
Genomic profile of carbapenem- and polymyxin-resistant *Klebsiella pneumoniae* isolates, showing AMR genes, plasmids, and virulence-associated genes.

The carbapenem- and polymyxin-resistant *K. pneumoniae* isolates were all found to belong to the sequence type 16 (ST16) with average nucleotide identity values of ≥99.99% (Table S1). The isolates harbored multiple antimicrobial resistance and virulence genes (Fig. 1), with a similar pattern of antimicrobial resistance (AMR) genes including the carbapenemase genes *bla*_NDM-1_, *bla*_OXA-9_, *bla*_OXA-10_, and *bla*_OXA-232_ (except isolate NT10 which lacked the *bla*_OXA-232_ gene) and other beta-lactamase genes (*bla*_CTX-M-15_, *bla*_SHV-1_, *bla*_TEM-1A_, and *bla*_VEB-1_). In addition, genes mediating resistance to aminoglycoside, fluoroquinolones, rifampicin, chloramphenicol, trimethoprim, macrolides, sulfisoxazole, and tetracycline were found in all isolates. Virulence genes harbored in the isolates included fimbriae and capsular genes, efflux pump genes AcrAB, genes involved in the regulation of capsular polysaccharide biosynthesis RcsAB, and genes of the type 6 secretion system involved in bacterial competitive advantage and associated with pathogenesis and biofilm formation. Plasmid profiling showed the presence of similar plasmids including the Col440I, Col440II, ColKP3, IncA/C2, IncFIA, and IncFIB(pQil). However, the plasmid ColKP3 was not found in isolate NT10, whereas the plasmid Col440II was not found in isolate NT23. The bla_OXA-232_ gene and ColKP3 plasmid were identified in the same contig; thus, ColKP3 plasmid might be harboring the *bla*_OXA-232_ gene.

### Molecular characteristics of polymyxin resistance

Polymyxin resistance-related genes were compared to the complete sequence of a polymyxin-susceptible *K. pneumoniae* MGH 78578 strain (NC_009648.1) (24). The results demonstrated the presence of mutations resulting in amino acid substitutions in multiple genes. Table 2 presents nucleotide changes that resulted in an amino acid change (missense and nonsense mutations). However, nucleotide changes without amino acid change (silent mutation) were not reported in this study. The effects of protein variants were predicted by the PROVEAN score. Most of the amino acid substitutions with PROVEAN score greater than −2.5 indicated missense and nonsense mutations, which did not affect the functionality of the protein and were interpreted as neutral. Two substitutions were observed in the *crrB* of the CrrAB two-component regulatory system which normally modulates the PmrAB system and is involved in modifications of lipopolysaccharide. The substitution of glutamine with leucine at position 180 (Q180L) indicated a PROVEAN score of −6.578 and was interpreted as a deleterious effect, whereas the substitution of cysteine with serine at position 68 (C68S) yielded a PROVEAN score of 2.234 and was interpreted as a neutral effect.

**TABLE 2:** Genomic alterations in polymyxin resistance-related genes^*c*^

Gene	Role	Mutation found in isolates^*a*^				
		% Identity	Nucleotide substitution^*c*^	Amino acid substitution	Prediction of protein function	
					PROVEAN score	Interpretation^*b*^
*acrB*	MDR efflux pump (25 – 27)	99.7	ND^*d*^	ND	ND	ND
*arnA*	Biosynthesis of undecaprenyl phosphate-α-L-Ara4N and transfer of the L-Ara4N moiety to lipid A (28)	99.0	GCT → TCC	A18S	−1.07	Neutral
			CTC → ATC	L260I	−0.045	Neutral
*arnB*		99.0	ATC → GTC	I100V	0.564	Neutral
			GAC → GCA	D112A	−0.683	Neutral
*arnC*		99.8	ND	ND	ND	ND
*arnD*		99.3	ATA → GTA	I53V	−0.367	Neutral
*arnE*		99.7	ND	ND	ND	ND
*arnF*		99.5	GCT → GTG	A34V	0.147	Neutral
*arnT*		99.5	ATG → CTG	M114L	0.942	Neutral
			GTT → ATT	V117I	−0.037	Neutral
			AGG → AAG	R372K	−0.371	Neutral
*crrA*	Two-component regulatory system CrrAB modulates the PmrAB system, and involved in modifications of lipopolysaccharide (29)	99.9	ND	ND	ND	ND
*ccrB*		99.5	TGT → AGT	C68S	2.234	Neutral
			CAA → CTA	Q180L	−6.578	Deleterious
*eptA*	Catalyzes the addition of a phosphoethanolamine moiety to the lipid A. The phosphoethanolamine modification is required for resistance to polymyxin (30).	99.7	ATC → GTC	I138V	−0.417	Neutral
*eptB*		99.6	GCG → GAG	A429E	2.605	Neutral
*kpnE*	Major facilitator superfamily efflux pump involved in broad-spectrum antimicrobial resistance (31, 32)	100	ND	ND	ND	ND
*kpnF*		100	ND	ND	ND	ND
*lpxM*	Catalyzes the transfer of myristate from myristoyl-acyl carrier protein to Kdo2-(lauroyl)-lipid IV(A) to form Kdo2-lipid A.	98.9	AGC → GGC	S253G	0.587	Neutral
*mgrB*	Small transmembrane protein produced upon activation of the PhoPQ signaling system and acts as a negative regulator of the PhoPQ system (33)	100	ND	ND	ND	ND
*pagP*	Catalyze palmitate transfer from a phospholipid to a glucosamine unit of lipid A	100	ND	ND	ND	ND
*phoP*	The two-component global regulatory system that plays role in antibiotic susceptibility, physiology, stress adaptation, and virulence (34)	99.7	ND	ND	ND	ND
*phoQ*		99.6	ND	ND	ND	ND
*pmrA*	The two-component regulatory system that modulates the addition of aminoarabinose to lipid A and confers resistance to cationic antimicrobial peptides	99.7	GAA → GGA	E57G	−1.762	Neutral
*pmrB*		99.8	ACC → GCC	T246A	1.132	Neutral
*pmrC*	Inner membrane protein required for the incorporation of phosphoethanolamine into lipid A and mediates polymyxin resistance	99.7	ATC → GTC	I138V	−0.417	Neutral
*pmrD*	Controls the post-transcriptional activity of the PmrA–PmrB system	99.6	ND	ND	ND	ND
*sapA*	ABC transporter periplasmic-binding protein system (35), involved in resistance to antimicrobial peptides	99.8	ATC → CTC	I65L	0.267	Neutral
*sapB*		99.7	ND	ND	ND	ND
*sapC*		99.6	ND	ND	ND	ND
*sapD*		99.6	ND	ND	ND	ND
*sapF*		99.8	ND	ND	ND	ND

^*a*^ The variants of nucleotide and protein substitutions were equally found in all the isolates.

^*b*^ If the PROVEAN score is less than or equal to −2.5, the protein variant is predicted to have a “deleterious effect.” If the PROVEAN score is greater than −2.5, the protein variant is predicted to have a “neutral effect.”

^*c*^ We only reported nucleotide changes that resulted in the amino acid change (missense and nonsense mutations), while the nucleotide changes that did not lead to the amino acid change (silent mutation) were not reported in this study.

^*d*^ ND, not detected.

### Antibacterial effect of combinations of polymyxin and vancomycin

**Fig 2:**
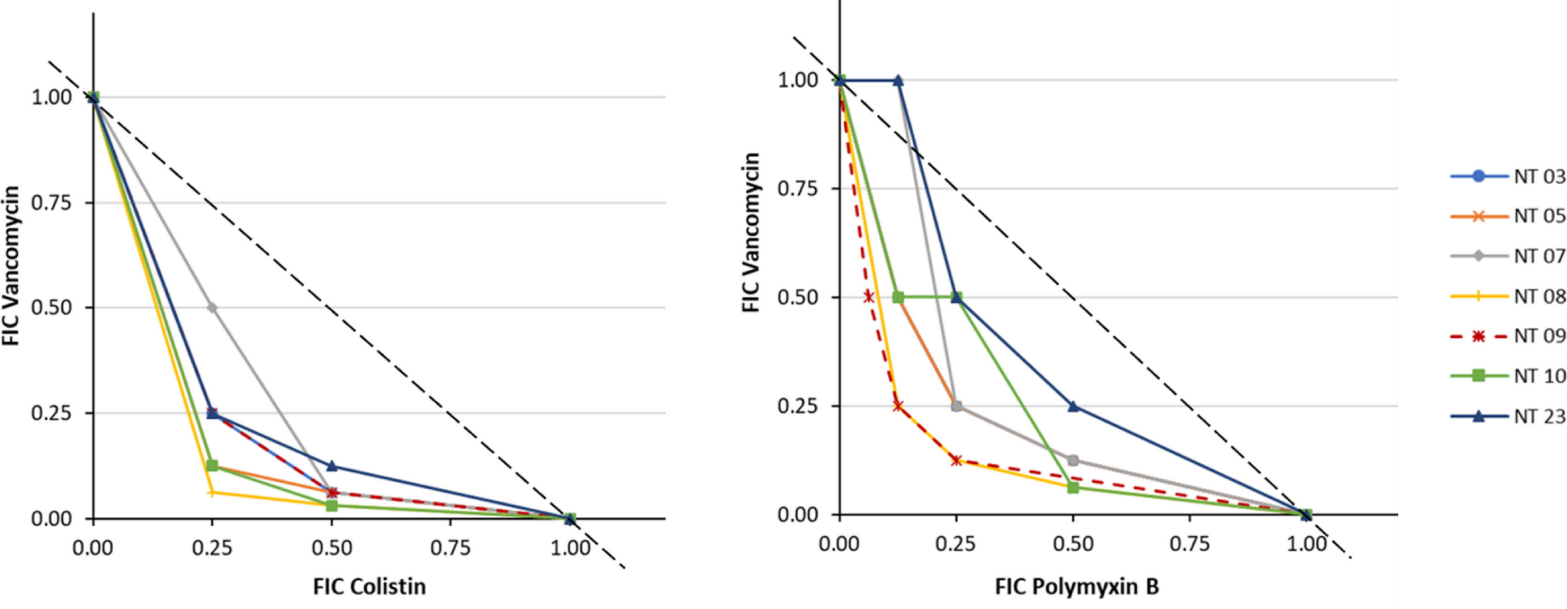
Isobologram of polymyxins and vancomycin combinations, showing superadditive against carbapenem- and polymyxin-resistant *Klebsiella pneumoniae* isolates.

The fractional inhibitory concentration index (FICI) (Table 1) presents the effects of combinations of polymyxin (B and E) with vancomycin. The results revealed FICI ranges of 0.38–1.13 for vancomycin with polymyxin B combinations and 0.31–1.13 for vancomycin with colistin combinations. Furthermore, the isobologram of fractional inhibitory concentrations (FICs) (Fig. 2) showed superadditive effects indicated by the downward concavity of the isoboles. Nonlinear isobolograms are used to indicate whether the effect produced by the combination of two drugs is synergistic (superadditive) or antagonistic (subadditive). From the isobole model, FICI = 1 represents an additive effect, FICI <1 represents a synergistic effect, whereas FICI >1 represents an antagonistic effect. The isobologram of combinations of vancomycin with polymyxin B and colistin showed that vancomycin in combination with either colistin or polymyxin B induced a synergistic effect against the isolates.

### Population analysis profiling of carbapenem- and polymyxin-resistant *K. pneumoniae*

**Fig 3:**
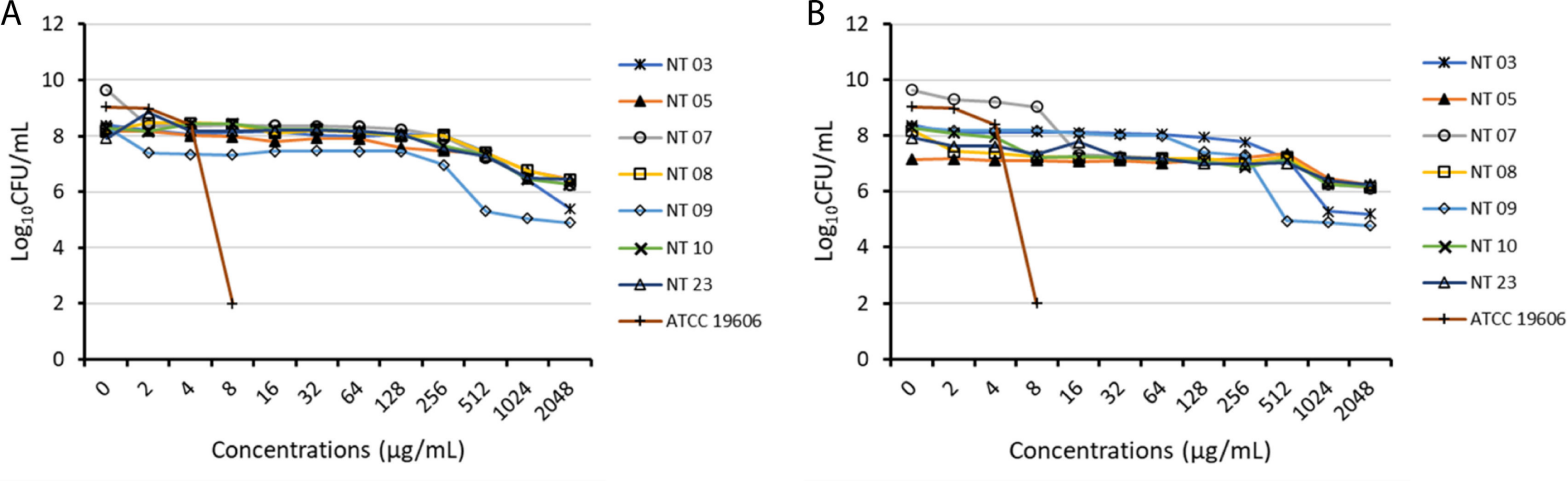
Result of population analysis profiling of carbapenem- and polymyxin-resistant *Klebsiella pneumoniae* for colistin (**A**) and polymyxin B (**B**).

**Fig 4:**
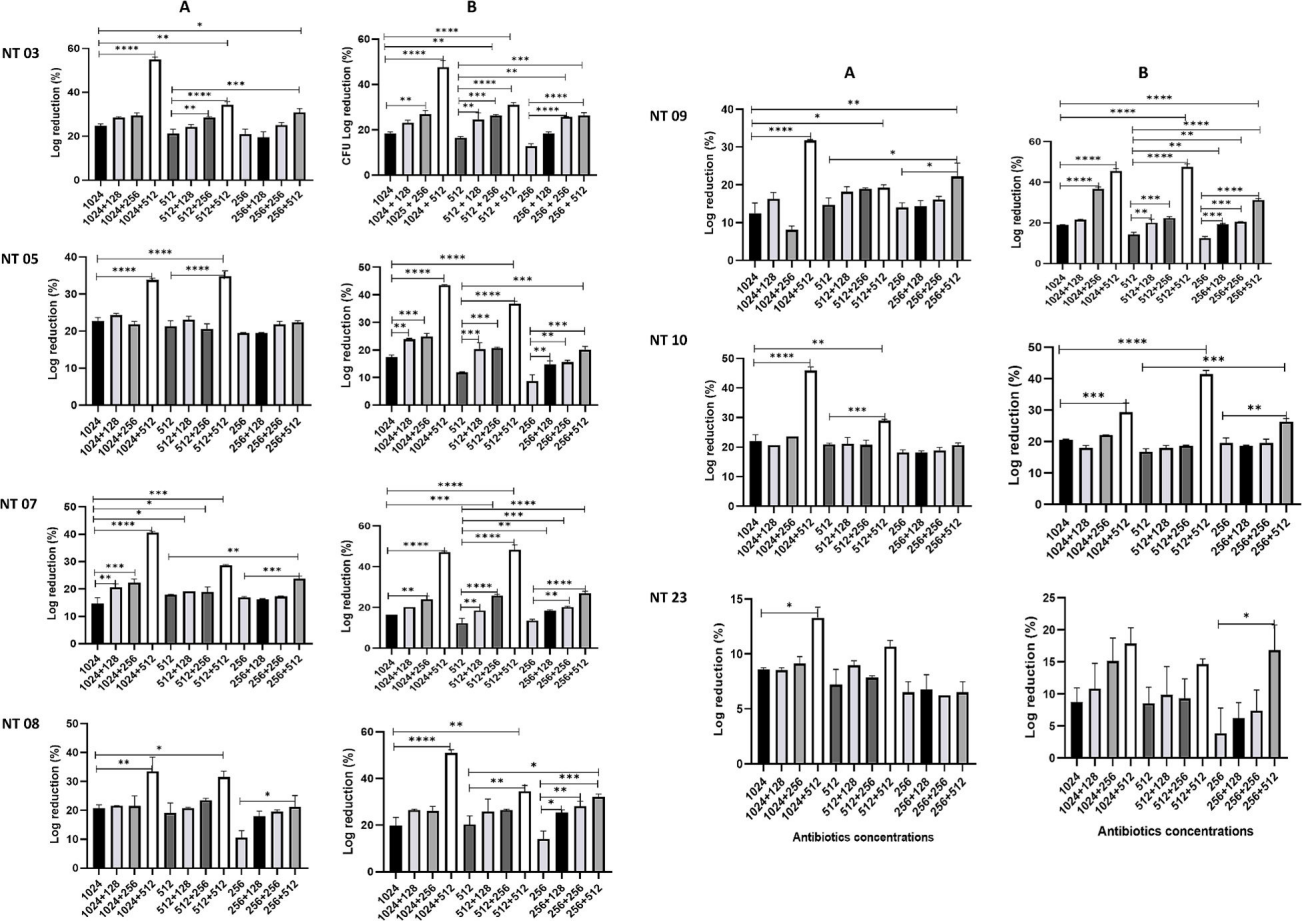
Result of population analysis profiling of the isolates on combinations of colistin and vancomycin (**A**) and polymyxin B and vancomycin (**B**).

The population analysis profiling of the isolates showed resistance to both colistin and polymyxin B, with the growth of subpopulations at 2,048 µg/mL (Fig. 3). Isolates NT03 and NT09 indicated heteroresistance to colistin and polymyxin B, with subpopulations with MIC of 256 µg/mL, while isolates 07 showed heteroresistance to polymyxin B, with subpopulations with MIC of 8 µg/mL. The population analysis profiling of dual antibiotics (colistin and vancomycin or polymyxin B and vancomycin) (Fig. 4) showed that at concentrations <2,048 µg/mL, combinations at sub-inhibitory concentrations demonstrated enhanced antibacterial activities with higher reduction in the growth.

### Time-kill kinetics of vancomycin combination with colistin or polymyxin B

**Fig 5:**
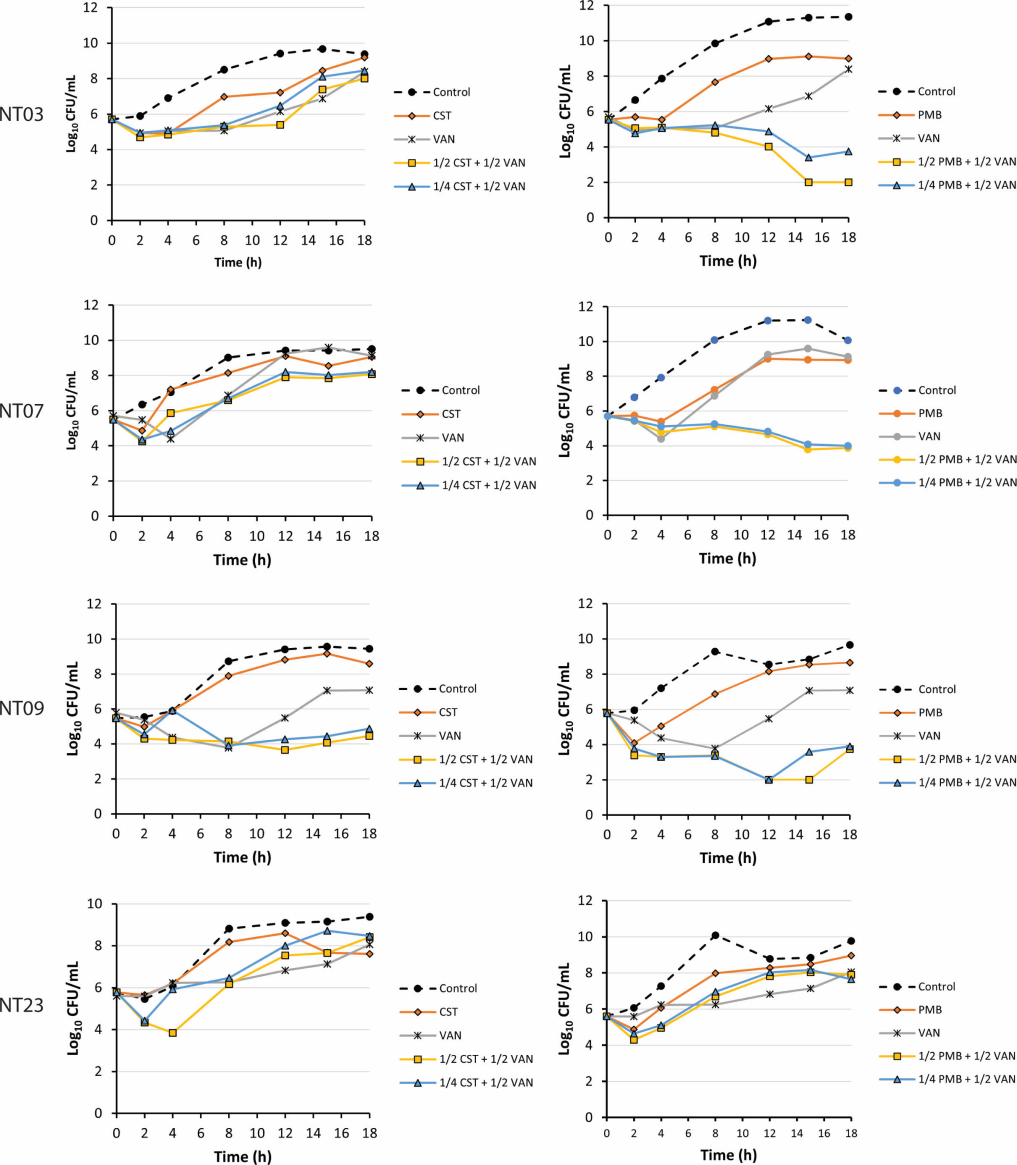
Time-kill graph of polymyxin and vancomycin combinations on carbapenem- and polymyxin-resistant *Klebsiella pneumoniae* isolates.

The time-kill kinetics of polymyxins in combination with vancomycin is presented in Fig. 5. The results demonstrated enhanced synergistic activity for polymyxin B and vancomycin combinations compared to colistin and vancomycin combinations. At 1/2MIC polymyxin B and 1/2MIC vancomycin, a >3 log reduction in colony-forming units per milliliter (CFU/mL) was recorded for isolate NT03 at 15 and 18 h, compared to cell count at 0 h. At 1/4MIC polymyxin B and 1/2MIC vancomycin, >2 log reduction in CFU/mL was observed. Similarly, isolate NT07 at 1/2MIC polymyxin B and 1/2MIC vancomycin displayed >2 log reduction, and at 1/4 MIC polymyxin B and 1/2 MIC vancomycin, showed >1 log reduction. At 12 h, a >3 log reduction in CFU/mL was observed for NT09; however, at 18 h, a regrowth was observed. At the tested concentrations, the combinations did not show effects for isolate NT23. Also, for isolates NT03, NT07, and NT23, combinations of colistin with vancomycin at the tested concentration did not provoke enhanced activities. However, for isolates NT09, the combinations demonstrated >1 log reduction in CFU/mL at 8 h and with subsequent regrowth after 12 h. The overall result demonstrated that combinations of polymyxins with vancomycin were effective against the isolates.

### Protein profiling analysis related to polymyxin and vancomycin activities

**Fig 6:**
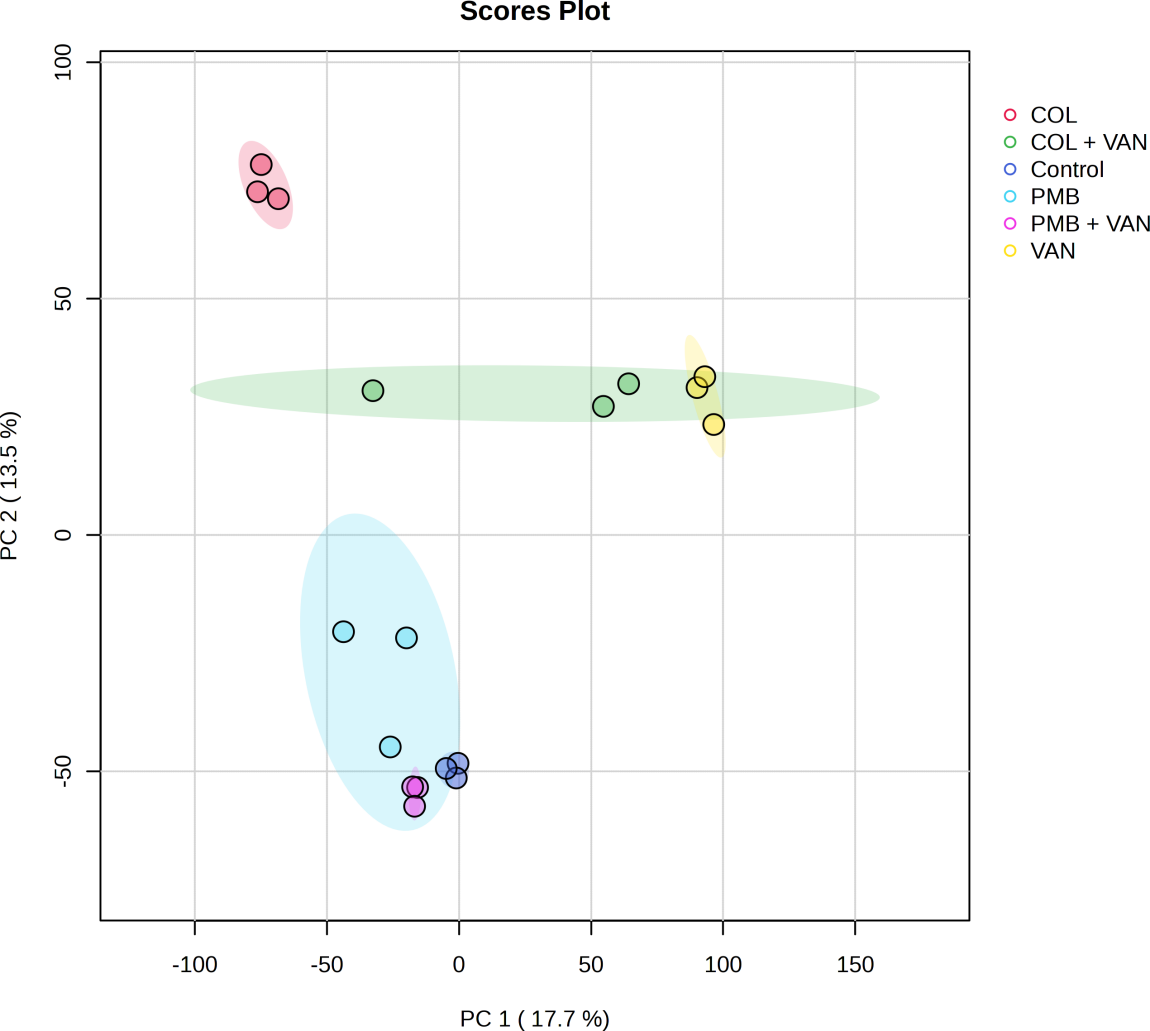
A 2D scores plot from a principal component analysis of all detected proteins demonstrating the global trends in differences across treatments. CST, colistin; PMB, polymyxin B; VAN, vancomycin; PC, principal component.

**Fig 7:**
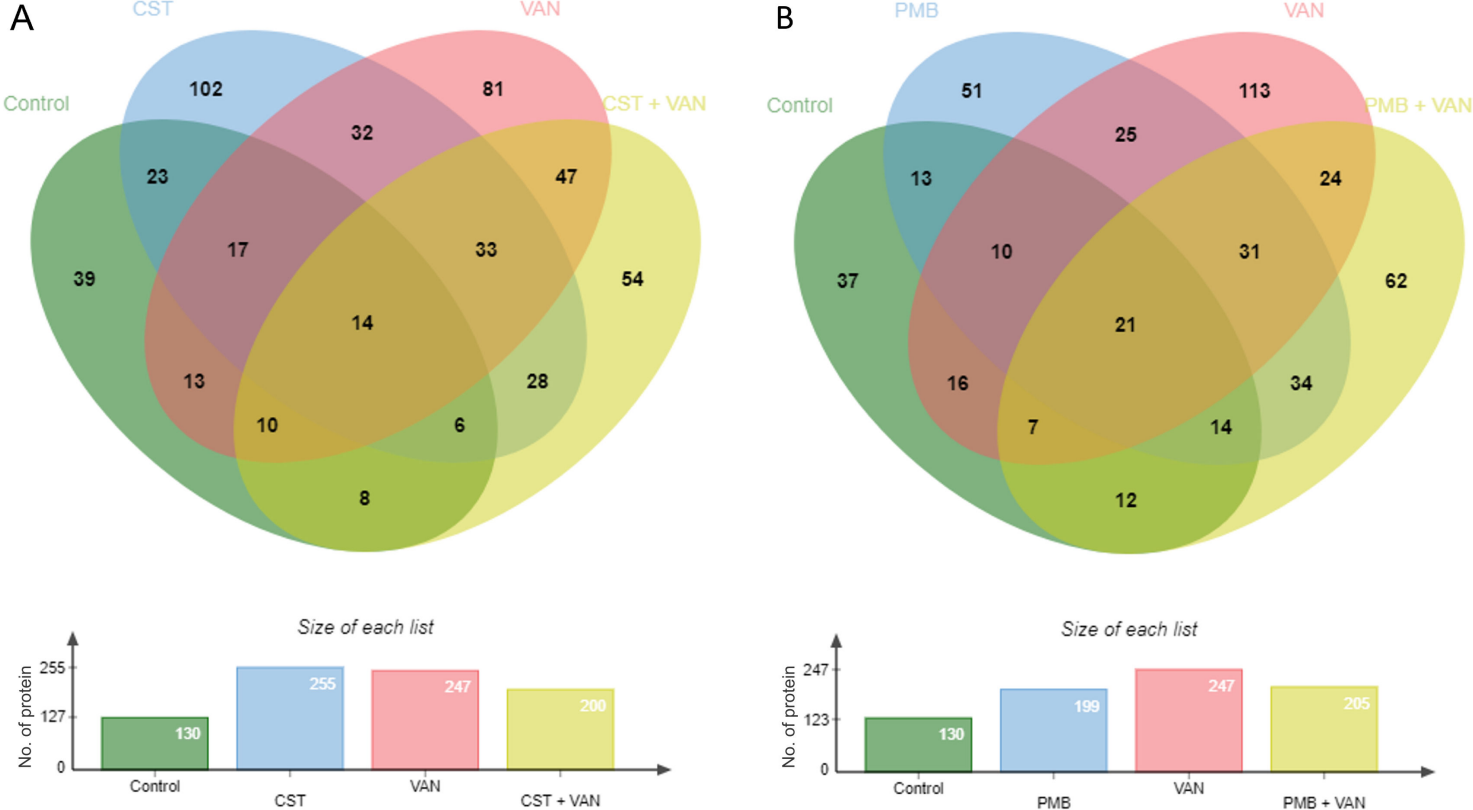
Venn diagrams of the detected proteins in the comparison among control, colistin, vancomycin, and colistin plus vancomycin (**A**) and the comparison among control, polymyxin B, vancomycin, and polymyxin B plus vancomycin (**B**). CST, colistin; PMB, polymyxin B; VAN, vancomycin.

**Fig 8:**
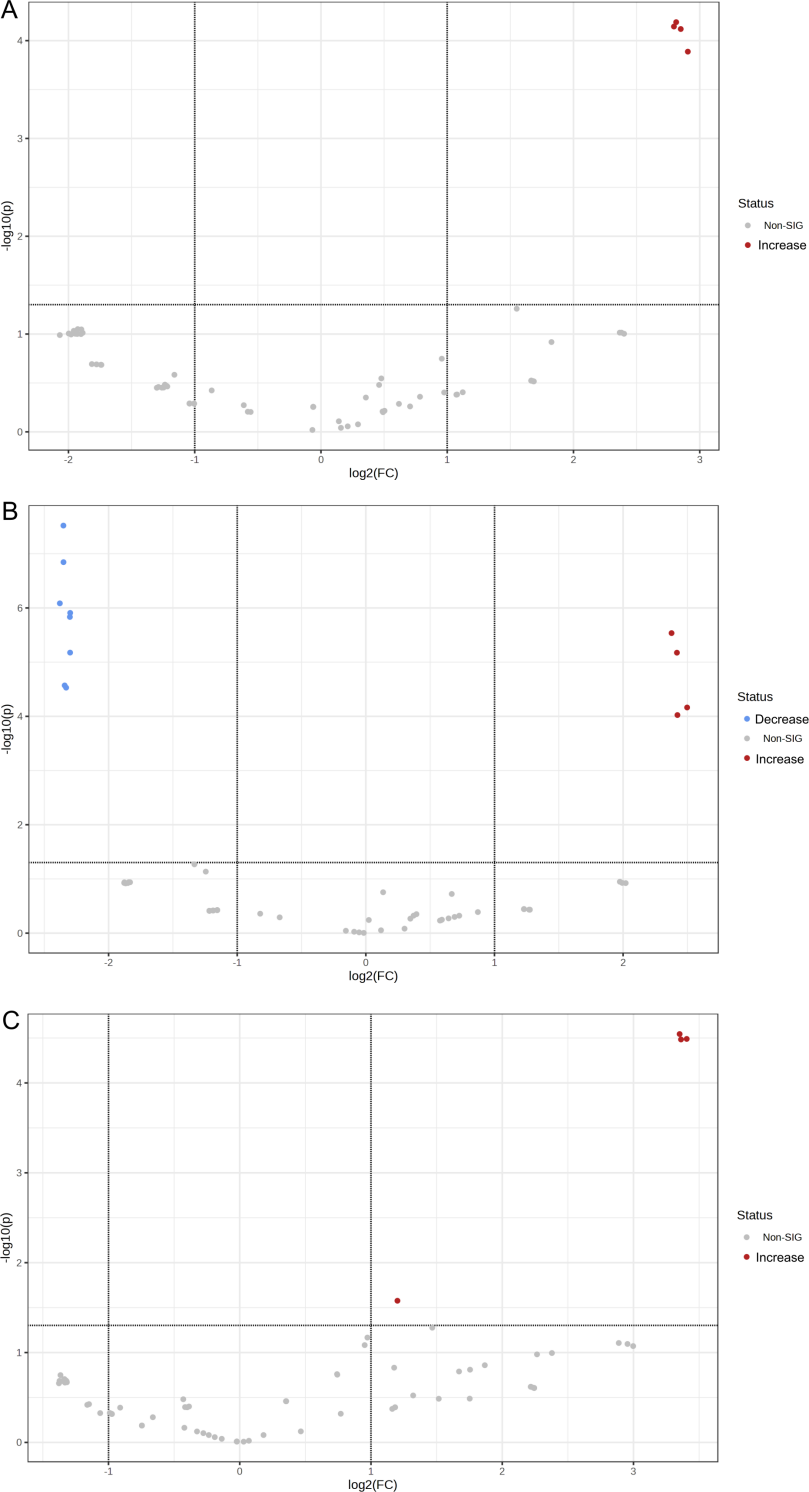
Volcano plots of differentially produced proteins (DPPs) in colistin (**A**), polymyxin B (**B**), and vancomycin (**C**) treatments, compared to the control group.

**Fig 9:**
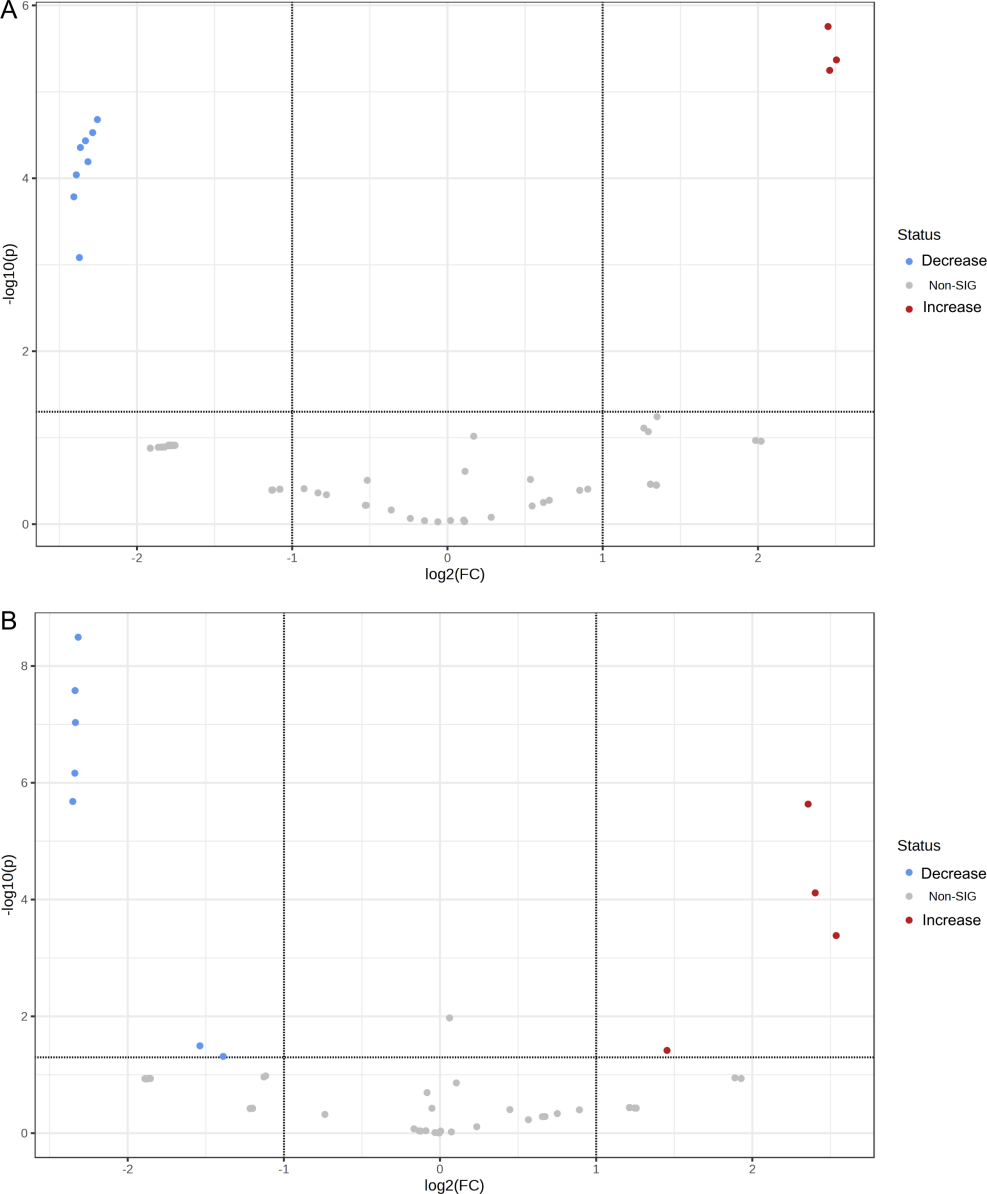
Volcano plots of differentially produced proteins (DPPs) in colistin plus vancomycin (**A**) and polymyxin B plus vancomycin (**B**) treatments, compared to the control group.

**Fig 10:**
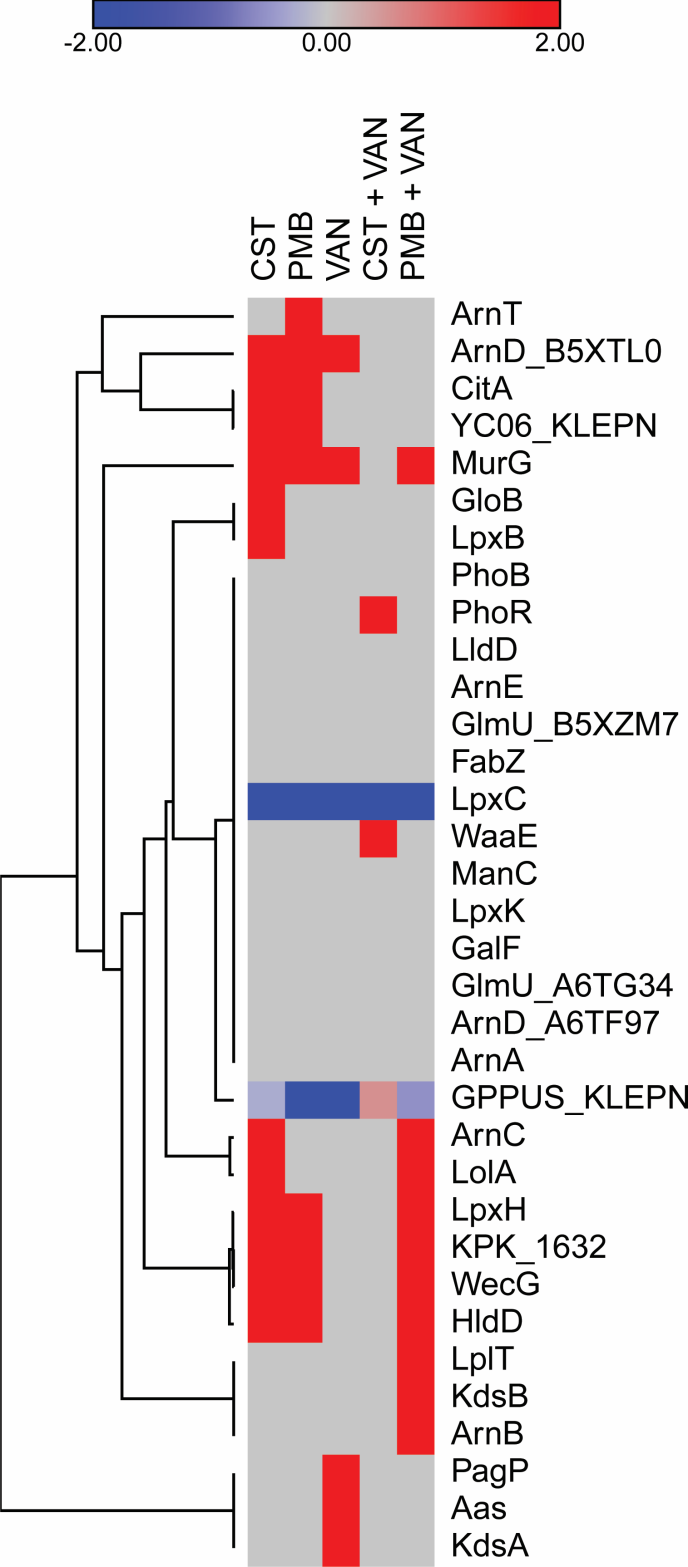
Heatmap of DPPs that might be associated with the activities of colistin, polymyxin B, vancomycin, colistin plus vancomycin, and polymyxin B plus vancomycin treatments, compared to the control group. CST, colistin; PMB, polymyxin B; VAN, vancomycin.

Isolate NT09 was used as a representative for this experiment because it was the only isolate that seems to show a response to colistin and polymyxin from the time-kill result. Protein profiling results of treatment and non-treatment groups are illustrated in Fig. 6 to 10 and Tables S2 to S5. In proteomes of six groups, the principal component analysis showed obviously separated groups indicating that proteomes were significantly changed among the groups. Venn diagram in Fig. 7A with the data in Table S3 revealed that 39 (7.69%), 102 (20.12%), 81 (15.98%), and 54 (10.65%) out of 507 proteins were only observed in the groups of control, colistin, vancomycin, and colistin plus vancomycin, respectively. A total of 33 (6.51%) proteins were shared among these treatment groups, whereas they were not found in the control group. Thirty-two (6.31%) proteins were detected in both groups of single-drug treatments (colistin and vancomycin), which were not presented in a group of combined drug treatment. Meanwhile, a Venn diagram in Fig. 7B with the data in Table S4 showed that 37 (7.87%), 51 (10.85%), 113 (24.04%), and 62 (13.19%) out of 470 proteins were only observed in the groups of control, polymyxin B, vancomycin, and polymyxin B plus vancomycin, respectively. A total of 31 (6.60%) proteins were shared among these treatment groups, whereas they were not found in the control group. Twenty-five (5.32%) proteins were detected in both two groups of single-drug treatment (polymyxin B and vancomycin), which were not presented in a group of combined drug treatment.

In addition, volcano plots showing differentially produced proteins (DPPs) in all treatment groups (*n* = 5) and control groups are presented in Fig. 8 and 9; Table S5. Among the proportion of statistical significance of DPPs, four different proteins showed increased abundance in the colistin, polymyxin B, vancomycin, and polymyxin B plus vancomycin groups, while three proteins showed increased abundance in the colistin plus vancomycin treatment group, respectively. Similarly, eight proteins showed decreased abundance in the polymyxin B and the colistin plus vancomycin groups, and seven proteins, for the polymyxin B plus vancomycin, while no proteins showed decreased abundance in the colistin and the vancomycin treatment groups, respectively, compared to the control group. Remarkably, the MdtJ protein was significantly increased and the DsbD protein was significantly decreased in only groups of combined drug treatments.

Among the 796 proteins found among five treatment groups and the control group, 34 (4.27%) proteins involved in polymyxins and vancomycin activities were selected, and their fold changes are shown in Fig. 10. Hierarchical clustering of these 24 proteins revealed two groups (*n* = 3 and *n* = 31). The PhoR and WaaE proteins were increased in the colistin plus vancomycin treatment group, while the LplT, KdsB, and ArnB proteins were increased in the polymyxin B plus vancomycin treatment group, compared to the control group. Additionally, increase of the ArnT protein in polymyxin B and the PagP, Aas, and KdsA proteins in vancomycin was observed.

## DISCUSSION

The resistance of *K. pneumoniae* to polymyxins limits treatment options for infections caused by carbapenem resistance isolates and has become a global health emergency. The whole-genome sequencing showed that all the polymyxin-resistant isolates belonged to ST16 and shared similar genomic composition (≥99.99% identity) (36). This might be because all the isolates were sourced from an intensive care unit of the same hospital and might point to the local spread of isolates from the same clone within the facility. Antimicrobial resistance gene profiling revealed several resistant mediating genes responsible for multidrug resistance. A similar occurrence of multiple AMR genes especially of extended-spectrum beta-lactamases (ESBL) and carbapenemase genes in *K. pneumoniae* isolates has been rampantly reported (37, 38). Due to the high prevalence and the intraspecies spread of AMR genes in *K. pneumoniae*, it has been described as a major worldwide source and shuttle for antibiotic resistance and a key trafficker of drug resistance genes from environmental to clinically important bacteria (39, 40). The isolates carried genes encoding for carbapenems, aminoglycoside, fluoroquinolones, rifampicin, chloramphenicol, trimethoprim, macrolides, sulfisoxazole, and tetracycline resistance, in addition to beta-lactamase genes including *bla*_NDM-1_, *bla*_OXA-10_, *bla*_OXA-232_, and *bla*_OXA-9_. The isolates also harbored fimbriae and capsular genes, efflux pump genes, capsular polysaccharide biosynthesis genes RcsAB, and genes of the type 6 secretion system coding for virulent factors. Furthermore, plasmid types were identified in the isolates, especially the ColKP3 plasmid with the carriage of the class D carbapenemase gene, *bla*_OXA-232_. Studies have previously reported that the *bla*_OXA-232_ gene is often harbored in the ColKP3 plasmid (41 – 43). Genomic profiling for polymyxin-resistant mediators identified the occurrence of multiple mutations resulting in amino acid substitutions. Amino acid substitutions in genes regulating lipid A biosynthesis, lipopolysaccharide biosynthesis, the two-component regulatory systems PhoQP, PmrAB, CrrAB, and the MgrB regulator are associated with polymyxin resistance. Although numerous mutations were present in the isolate, most of the mutations did not confer a deleterious effect on the gene function. However, an amino acid substitution in the CrrAB system resulted in a mutation in the *crrB* gene with a deleterious effect on the protein function. Deleterious effects resulting from mutations in the *crrB* gene have been reported as responsible for polymyxin resistance (29, 44 – 46). In addition, the presence of efflux pump genes including the *sapABCDF* operon associated with resistance to antimicrobial peptides (47), the *kpnEF* genes in a small multidrug resistance (SMR)-type efflux pump (31), and the *acrB* gene (25 – 27), which upon activation results in the increase of polymyxin resistance, might likewise contribute to the resistance of the isolates.

The use of combination therapies is an interim strategy for the effective management of polymyxin-resistant isolates. Studies have evinced the promising synergistic effects of polymyxins in combination with antibiotics of other classes. We investigated the effects of polymyxin B and polymyxin E (colistin) combinations with vancomycin, as a drug repurposing strategy for the management of infections caused by Gram-negative bacteria. The combinations demonstrated better *in vitro* effects when compared to individual antibiotic monotherapy. Previous studies have demonstrated that adjunctive vancomycin therapies potentiate enhanced antimicrobial effects of polymyxins on MDR *A. baumannii* (20, 21, 48 – 50). However, the effects of these combinations on MDR *K. pneumoniae* isolates have not been elaborated. Although vancomycin is inactive against Gram-negative pathogens due to its large molecular size and inability to penetrate the outer bacterial membrane (51), compromise of bacterial outer membrane integrity by polymyxins might promote the penetration of vancomycin molecules through the outer membrane, resulting in synergistic bacteriostatic or bactericidal effects. Moreover, modification of vancomycin through conjugation with arginine conferred antimicrobial potentials against carbapenem-resistant *E. coli*, by targeting cell wall synthesis (52). The checkerboard assays indicated that the combination of polymyxins and vancomycin was not antagonistic but varied over a range of FICI, with synergistic, additive, or indifferent outcomes. Isobologram of FICs yielded a characteristic superadditive curve, with isobars below the additive line. Furthermore, the time-kill assay showed that the combinations exhibited higher bactericidal effects compared to individual monotherapies as demonstrated by the log reduction in CFU/mL. However, the combination of polymyxin B and vancomycin displayed better activities than combinations of colistin and vancomycin. It has been previously reported that polymyxin B exhibited better antimicrobial effects compared to colistin (53). Based on the pharmacokinetics of the drugs, polymyxin B achieves rapid therapeutic concentrations, provides more predictable serum concentrations, does not require renal dose adjustments, and might not necessitate a loading dose. In contrast, colistin is required to prolong time to achieve peak serum concentration, with unpredictable serum concentrations due to significant interpatient variability; renal dose adjustments must be considered; and a loading dose is required (54). Additionally, a recent systematic review and a prospective study on the nephrotoxicity of colistin and polymyxin B concluded that colistin is associated with significantly higher nephrotoxicity compared to polymyxin B (55, 56).

Recently, heteroresistance which is defined as the occurrence of subpopulations with elevated antimicrobial tolerance within a population has become a critical issue in antimicrobial susceptibility testing and is often associated with false-positive or false-negative antimicrobial susceptibility results. Thus, we further performed a population analysis profiling of the isolates on individual polymyxin antibiotic and dual antibiotics (colistin + vancomycin OR polymyxin B + vancomycin). The results revealed that the isolates were entirely resistant to both polymyxins, with no susceptible subpopulation. Enhancement in bacterial log reduction (%) to dual antibiotics at reduced concentration was observed compared to single polymyxins at higher concentrations. Furthermore, the results indicated that the polymyxin B and vancomycin combinations were superior to colistin and vancomycin combinations. Previous studies have demonstrated heteroresistant through population analysis profiling of resistant isolates to antibiotic combinations with additive outcomes (2, 57). Most studies attribute heteroresistance to the co-occurrence of susceptible and resistant subpopulations within a population. However, heteroresistance includes the co-occurrence of subpopulations with various levels of resistance within a population, as well as the co-occurrence of subpopulations with various levels of susceptibility within a population (58). This study indicates that novel mutation in the CrrB protein and the presence of efflux pump genes might be responsible for high-level polymyxin resistance and highlights that repurposing of vancomycin might benefit the antimicrobial activities of polymyxins against MDR *K. pneumoniae*.

In the proteomic analysis, where the expected number of proteins was approximately 5,300, the actual liquid chromatography-tandem mass spectrometry (LC-MS/MS) results in our study showed only 796 proteins. Factors contributing to this phenomenon may include sample complexity, limited LC-MS/MS range, instrument sensitivity, post-translational modifications, and data analysis criteria (housekeeping protein exclusion). Furthermore, our findings revealed that the protein levels in the control group were lower compared to the treatment groups. It is possible that not all *Klebsiella* cells were eradicated within 6 h of antibiotic exposure. As a result, we postulate that the surviving cells could have been highly active in defending against the antibiotics, leading to higher protein levels observed in the treatment groups. Protein profiling also revealed possible mechanisms of action of polymyxin and vancomycin combination, compared to each single-drug treatment and non-treatment. The functions of all the studied proteins were retrieved from the UniProt database (https://www.uniprot.org/ ), as illustrated in Table S2. A total of three to four increased protein abundance and zero to seven decreased protein abundance was found among the treatment groups, compared to the control group. The DPPs might be associated with polymyxin and vancomycin activities, since they were only detected in the treatment groups. In the comparison between single-drug treatments and combined drug treatments, the increased MdtJ protein and the decreased DsbD protein were only observed in polymyxin plus vancomycin combination. MdtJ is a protein belonging to the SMR family efflux pumps, initially associated with resistance against deoxycholate and sodium dodecyl sulfate (SDS) in *Escherichia coli* (59). We then hypothesize that the increased MdtJ production in our *Klebsiella pneumoniae* isolates can enhance antibiotic efflux, reducing susceptibility and promoting multidrug resistance, cross-resistance, persistent infections, and horizontal gene transfer of resistance genes (60). DsbD (thiol:disulfide interchange protein DsbD) generally transports electrons from cytoplasmic thioredoxin to the periplasmic oxidized substrates, which are involved in the formation and reshuffling of disulfide bonds in proteins. Decrease of the DsbD protein may have consequences for bacterial cell physiology, including the proper folding of proteins (especially outer membrane porin and efflux pumps), susceptibility to oxidative stress, and virulence (61). Therefore, decreased DsbD protein probably compromises the ability of bacteria to resist antibiotics, leading to increased sensitivity to polymyxin plus vancomycin combination.

The heatmap with selected protein features (Fig. 10; Table S2) revealed some increased protein abundance, which were specifically observed in combined drug treatments. In the colistin plus vancomycin treatment, increased PhoR and WaaE proteins were found. PhoR (phosphate regulon sensor protein PhoR) is a member of the two-component regulatory system PhoR/PhoB involved in the phosphate regulon genes expression, enhancing bacterial survival in low-phosphate environments. Meanwhile, WaaE [lipopolysaccharide (LPS) core biosynthesis glycosyltransferase WaaE] is involved in the biosynthesis of the LPS core, which is a component of the outer membrane of Gram-negative bacteria. We hypothesize that increase of the WaaE protein may lead to alterations in the LPS structure, reducing the binding of colistin to the bacterial cell surface and decreasing its effectiveness. In the polymyxin B plus vancomycin treatment, the LplT, KdsB, and ArnB proteins were increased. LplT [lysophospholipid (LPL) transporter LplT] generally catalyzes the facilitated diffusion of 2-acyl-glycero-3-phosphoethanolamine (2-acyl-GPE) into the cell, leading to changes in the composition of the bacterial cell membrane. Therefore, increase of the LplT protein may contribute to bacterial resistance to antimicrobial agents that target the cell membrane. KdsB (3-deoxy-manno-octulosonate cytidylyltransferase) generally catalyzes the transfer of a cytidine monophosphate group to 3-deoxy-D-manno-octulosonic acid, an important component of the LPS molecule. We then hypothesize that increase of the KdsB protein probably increases the production and the alteration of LPS, leading to antibiotic resistance in bacteria. ArnB (UDP-4-amino-4-deoxy-L-arabinose–oxoglutarate aminotransferase) is involved in the biosynthesis of LPS by attaching the modified arabinose to lipid A. Thus, we hypothesize that increase of ArnB may result in resistance to polymyxin B and cationic antimicrobial peptides.

## MATERIALS AND METHODS

### Culture media, antibiotics, and bacteria used in this study

All culture media used were purchased from Becton Dickinson & Co. Difco (Franklin Lakes, NJ, USA). Colistin sulfate salt and polymyxin B sulfate salt were obtained from Sigma-Aldrich (Saint Louis, MO, USA). Vancomycin (Vancin-S) was obtained from Siam Pharmaceuticals Co., Ltd., Bangkok, Thailand. The isolates were obtained from patients receiving treatment in Naradhiwas Rajanagarindra Hospital, Southern Thailand, and were preliminarily investigated for carbapenem resistance as described (7). *Acinetobacter baumannii* ATCC 19606 was used as quality control. All the bacterial cultures were stored in tryptic soy broth (TSB supplemented with 40% glycerol and kept at −80°C.

### Antibacterial susceptibility testing

MICs were determined by the broth microdilution method as recommended by the CLSI guidelines (23). Serial twofold dilutions of antibiotics were prepared in Mueller-Hinton broth, and 100 µL of 1 × 10^6^ CFU/mL bacterial suspension was added to 100-µL antibiotic in each well and incubated at 37°C for 18 h. MIC was observed using the resazurin test and recorded as the lowest concentration of the antibiotic without visible color change after 2 h of treatment.

### Whole-genome sequencing

Genomic DNA of seven carbapenem- and polymyxin-resistant *K. pneumoniae* isolates were extracted using TIANamp Bacteria DNA kit (Tiangen Biotech, Beijing Co., Ltd.) following the manufacturer’s protocol. The extracted DNA was sent to Beijing Genomics Institute (BGI) in China to perform short-read sequencing. DNA quality was checked using Qubit Fluorometer and Agarose Gel Electrophoresis. The qualified DNA was sequenced using the BGISEQ-500 platform (BGI, China). After receiving the results from BGI, the sequence reads were *de novo* assembled using Unicycler v0.4.7 (62). Then, the assembled genomes were annotation using Prokka v1.12 (63). The average nucleotide identity (ANI) among the seven genomes was analyzed using FastANI v1.32 (36), while the sequence types were identified using MLST 2.0 (https://cge.cbs.dtu.dk/services/MLST/) (64). AMR genes were detected using ResFinder 4.1 (https://cge.cbs.dtu.dk/services/ResFinder/), with a selection of ≥95% identity and ≥80% coverage (65). Also, plasmids were investigated using PlasmidFinder 2.1 (https://cge.cbs.dtu.dk/services/PlasmidFinder/), with a selection of ≥80% identity and ≥80% coverage (66). To explore mutation associated with colistin resistance, the reference sequences of genes were retrieved from Natural Center for Biotechnology Information (NCBI) (https://www.ncbi.nlm.nih.gov/). The reference sequences were then aligned with our genomes using Geneious R10.26 (67). Only nucleotide changes that resulted in an amino acid change were reported, and the effect of amino acid change on protein function was predicted using Protein Variation Effect Analyzer tool (PROVEAN) v.1.1.5 (http://provean.jcvi.org/index.php) (68). For the interpretation, if the PROVEAN score is less than or equal to −2.5, the protein variant is considered to have a deleterious effect, whereas if the PROVEAN score is greater than −2.5, the protein variant is considered to have a neutral effect (68, 69). In addition, virulence-associated genes were detected using blastn v2.12.0, with the identity and E-value cut-offs of 80% and 1e-10, respectively, against the virulence factor database of *Klebsiella* spp. (http://www.mgc.ac.cn/cgi-bin/VFs/genus.cgi?Genus = Klebsiella).

### Combination assay

The effects of vancomycin combination with either colistin or polymyxin were investigated using the checkerboard technique as previously modified (18). In brief, antibiotic dilutions containing 50 µL of serially diluted colistin or polymyxin B and 50 µL of serially diluted vancomycin were prepared in a 96-well plate. A 100 µL of 1 × 10^6^ CFU/mL bacterial suspension was added to each well, and the plates were incubated for 18 h at 37°C. Inhibitory concentrations were determined as concentrations without visible color changes as indicated by the resazurin test. The antibacterial effects of single antibiotics were tested as a control. The experiment was performed for three independent repeats. The effects of the antimicrobial combination were defined according to the FICI as shown in the following equation:


$$FICI=\frac{MIC\;of\;drug\;A\;in\;combination}{MIC\;of\;drug\;A\;alone}+\frac{MIC\;of\;drug\;B\;in\;combination}{MIC\;of\;drug\;B\;alone}$$

The FICI results for each combination were interpreted as follows: FICI ≤0.5, synergism; 0.5 < FICI < 1, additive; 1 ≤ FICI < 2, indifference; and FICI ≥2, antagonism.

### Population analysis profile of bacterial isolates

Population analysis profiling was used to investigate for colistin and polymyxin B heteroresistance (2), with slight modifications. Briefly, 100 mL of overnight bacterial culture in TSB was adjusted to 0.5 McFarland standard (10^8^ CFU/mL) in phosphate buffer solution. The cultures were serially diluted from 10^8^ to 10^1^, and dilutions were plated onto Mueller-Hinton agar containing a twofold gradient of various concentrations of colistin or polymyxin B from 2 to 2,048 µg/mL, respectively. The drop plate technique was employed in this study. After 24 h of incubation at 37°C, the subpopulations that grew on the plates were enumerated. Heteroresistance was defined as the presence of a subpopulation of cells capable of growing at a concentration of antibiotics at least twofold higher than that of the antibiotic-susceptible parental strains. The population analysis profile of the bacterial isolates was further conducted with antibiotic combinations of polymyxin and vancomycin. Single polymyxin concentrations of 256–1,024 µg/mL and combinations with subinhibitory concentrations at 128–512 µg/mL of vancomycin were prepared on Mueller-Hinton agar (MHA) plates. Exponential phase bacterial cultures incubated for 5–6 h in Mueller-Hinton broth (MHB) were adjusted to 10^8^ CFU/mL and diluted further to 10^7^, 10^6^, 10^5^, 10^4^, 10^3^, 10^2^, and 10^1^. Serial dilutions of bacterial culture were drop plated at each concentration of antibiotic and incubated at 37°C for 48 h. Antibiotic combination treatment was compared with treatment with polymyxin alone, and the results were presented as log reductions in CFU/mL.

### Time-kill assay

The antibiotic efficacy of colistin, polymyxin B, vancomycin, and combinations of either colistin or polymyxin B with vancomycin against the polymyxin-hyper-resistant isolates was evaluated by an *in vitro* time-kill assay. In brief, overnight cultures of each of the four selected isolates were diluted to 10^6^ CFU/mL and exposed to the MICs of each antibiotic and combinations of sub-inhibitory concentrations in MHB. Viable cells were enumerated at 0, 2, 4, 8, 12, 15, and 18 h by spot plating and expressed in CFU/mL. The limit of detection was set at 100 CFU/mL.

### Protein profiling

To assess the possible mechanisms of the monotherapy and combination therapy against carbapenem- and polymyxin-resistant *K. pneumoniae* isolates, protein profiling was investigated using LC-MS/MS. Briefly, a representative isolate was selected and treated with colistin, polymyxin B, vancomycin, colistin plus vancomycin, and polymyxin B plus vancomycin at 37°C and 150 rpm for 6 h, while a non-treatment group was used as a control. The cultures were centrifuged at 4°C and 10,000 rpm for 10 min, and the supernatants were discarded. The pellets were resuspended in 800 µL of 1× phosphate-buffered saline and 200 µL of 10% SDS. They were incubated at 37°C for 1 h and then sonicated on ice with the condition of 30%, 9 pulse, 95 Aml, and 15 min. Then, they were centrifuged at 4°C and 12,000 rpm for 15 min, and the supernatants were collected. The protein concentration was measured using the Bradford assay, and the concentrations were normalized. Normalized protein concentrations were then sent to the National Center for Genetic Engineering and Biotechnology for performing the LC-MS/MS.

Protein quantitation was assessed by Maxquant 1.6.6.0. The analyzed LC-MS/MS data from Maxquant 1.6.6.0 were searched against the Uniprot *Klebsiella pneumoniae* database using the Andromeda software for protein identification. For all comparison, the data sets of statistically significant proteins (*P* < 0.05) from the LC-MS/MS results were statistically analyzed by analysis of variance (ANOVA) with Fisher’s Least Significant Difference (LSD) test. Then, all DPPs were analyzed for their intersections among the different sample groups using jvenn (http://jvenn.toulouse.inra.fr/app/example.html) (70). To identify the fold changes of DPPs with statistical significance, the volcano plots were generated using MetaboAnalyst 5.0 (https://www.metaboanalyst.ca/). Generating volcano plots in MetaboAnalyst 5.0 involves uploading and preprocessing metabolomics data, normalizing by medium, applying statistical tests to detect DPPs between groups, and plotting proteins on a graph based on statistical significance (*P*-value) and fold change (effect size) (71). In addition, the heatmaps of the proteins related to polymyxin and vancomycin activities were also constructed using MetaboAnalyst 5.0.

### Statistical analysis

All experiments were performed in triplicate for two independent repeats. Results were presented as mean ± standard deviation. Statistical analysis was performed using GraphPad Prism v8. Comparisons between means were carried out using analysis of variance and interpreted based on Tukey multiple comparisons at *P* < 0.0001. For protein profiling, the statistics in the comparison between treatment and control groups were performed by ANOVA with Fisher’s LSD test at *P* < 0.05.

## Data Availability

The assembled sequences of all seven polymyxin-resistant *K. pneumoniae* isolates have been deposited in NCBI under BioProject number PRJNA798670 with BioSample numbers SAMN25118149 to SAMN25118155. GenBank accession numbers GCA_023546055.1, GCA_023546045.1, GCA_023546015.1, GCA_023545985.1, GCA_023545965.1, GCA_023545935.1, and GCA_023545905.1 were assigned for the NT03, NT05, NT07, NT08, NT09, NT10, and NT23 genomes. The MS/MS raw data and analysis files have been deposited in the ProteomeXchange Consortium (http://proteomecentral.proteomexchange.org) via the jPOST partner repository (https://jpostdb.org) with the data set identifiers JPST002333 and PXD045685.

## SUPPLEMENTAL MATERIAL

Tables S1 to S5 spectrum.01199-23-s0001.xlsx This excel file contained Tables S1 to S5.

ASM does not own the copyrights to Supplemental Material that may be linked to, or accessed through, an article. The authors have granted ASM a non-exclusive, world-wide license to publish the Supplemental Material files. Please contact the corresponding author directly for reuse.
